# Use of Supervised Machine Learning for GNSS Signal Spoofing Detection with Validation on Real-World Meaconing and Spoofing Data—Part II ^†^

**DOI:** 10.3390/s20071806

**Published:** 2020-03-25

**Authors:** Silvio Semanjski, Ivana Semanjski, Wim De Wilde, Sidharta Gautama

**Affiliations:** 1 Department of Communication, Information, Systems & Sensors, Royal Military Academy, 1000 Brussels, Belgium; 2 Department of Industrial Systems Engineering and Product Design, Ghent University, 9052 Ghent, Belgium; ivana.semanjski@ugent.be (I.S.); sidharta.gautama@ugent.be (S.G.); 3 Industrial Systems Engineering (ISyE), Flanders Make, Ghent University, 9052 Ghent, Belgium; 4 Septentrio N.V., 3000 Leuven, Belgium; wim.dewilde@septentrio.com

**Keywords:** global navigation satellite system, spoofing, meaconing, support vector machines, principal component analysis, model validation, safety-of-life, position-navigation-timing, federated learning, GPS, GNSS, PNT, SVM, SoL

## Abstract

Global Navigation Satellite System (GNSS) meaconing and spoofing are being considered as the key threats to the Safety-of-Life (SoL) applications that mostly rely upon the use of open service (OS) signals without signal or data-level protection. While a number of pre and post correlation techniques have been proposed so far, possible utilization of the supervised machine learning algorithms to detect GNSS meaconing and spoofing is currently being examined. One of the supervised machine learning algorithms, the Support Vector Machine classification (C-SVM), is proposed for utilization at the GNSS receiver level due to fact that at that stage of signal processing, a number of measurements and observables exists. It is possible to establish the correlation pattern among those GNSS measurements and observables and monitor it with use of the C-SVM classification, the results of which we present in this paper. By adding the real-world spoofing and meaconing datasets to the laboratory-generated spoofing datasets at the training stage of the C-SVM, we complement the experiments and results obtained in Part I of this paper, where the training was conducted solely with the use of laboratory-generated spoofing datasets. In two experiments presented in this paper, the C-SVM algorithm was cross-fed with the real-world meaconing and spoofing datasets, such that the meaconing addition to the training was validated by the spoofing dataset, and vice versa. The comparative analysis of all four experiments presented in this paper shows promising results in two aspects: (i) the added value of the training dataset enrichment seems to be relevant for real-world GNSS signal manipulation attempt detection and (ii) the C-SVM-based approach seems to be promising for GNSS signal manipulation attempt detection, as well as in the context of potential federated learning applications.

## 1. Introduction

To protect Global Navigation Satellite System (GNSS) information from spoofing and meaconing, one can employ two different protection layers: data-level protection by means of the Navigation Message Authentication (NMA), and signal-level protection (authentication) by using encryption of signal codes. Signal-level protection is currently employed for the Global Positioning System (GPS) Precise Positioning Service (PPS) with L1 and L2 P(Y) code ranging signals, the GALILEO Public Regulated Service (PRS) with authorized access to PRS E1A and E6A code signals, and the forthcoming GALILEO High Accuracy Service (HAS) with encrypted (E6-B) and pilot (E6-C) signal components. Although signal-level protection provides essential protection from spoofing, those signals are still vulnerable to the meaconing type of receive and replay attack [1,2,3]. Furthermore, those services employing NMA [4,5] such as GALILEO Open Service (OS-NMA) and Commercial Service Authentication (CAS) are essentially not providing protection from the meaconing type of receive and replay attack [1,2,3], and have limited protection from certain types of spoofing attacks (e.g., the spoofer’s ability to broadcast valid navigation messages) [6]. Both direct meaconing (utilizing beforehand processing of the authentic signal, extraction of its parameters, followed by its modified replay delay) and indirect meaconing attack (utilizing reception of authentic signals from different satellites by an antenna array, followed by their replay with different relative delays) is claimed to be particularly effective even against signal-level protection [7]. Therefore, Safety-of-Life (SoL) and the critical infrastructure’s GNSS users being aware of both meaconing (non-manipulated delayed GNSS information) and spoofing (intentionally manipulated GNSS information) is of utmost importance. 

In this manuscript, we further explore the possibility of applying a supervised machine learning-based approach to detect GNSS signal manipulation attempts, particularly spoofing and meaconing attempts. The motivation behind employing the supervised machine learning algorithms for detection of GNSS signal spoofing and meaconing, in particular, a C-Support Vector Machine (SVM) classification we introduce in this paper, lies in the fact that, at GNSS user receiver level, a number of measurements and observables are generated. The correlation patterns within those data can be established and monitored with the purpose of distinguishing between false and authentic GNSS measurements and observables. In addition, the application of an SVM classifier to GNSS spoofing detection has been previously demonstrated as promising by [8,9,10,11].

As was described in depth in Part I of this publication [11], various datasets for the C-Support Vector Machine model building and its validation have been used. As in Experiments I and II [11], all three groups of GNSS datasets are also used in Experiments III and IV. 

These are: 

(i) The laboratory spoofing dataset synthetically generated (simulated) in the laboratory;

(ii) The real-world meaconing dataset recorded during a real-world meaconing event (unintentional re-radiation of GNSS signal). In the context of validation, this dataset is titled the meaconing validation dataset;

(iii) The real-world spoofing dataset recorded during a real-world spoofing event (intentional spoofing signal generated and radiated over the air). In the context of validation, this dataset is titled as spoofing validation dataset.

Terminology wise, we define GNSS signals in our work as either non-manipulated (authentic) or manipulated (false). Both non-manipulated (authentic) and manipulated (false) signal records are contained in each of the above listed datasets. 

In Part I [11], Experiments I and II were conducted by using the synthetically generated spoofing dataset for training dataset formation followed by the C-SVM model building, with eventual validation of the model by the meaconing validation dataset (Experiment I) and spoofing validation dataset (Experiment II).

Results of applying C-SVM in the Experiments I and II [11] showed that a relatively high number of supporting vectors has been involved in the separation between the authentic and false GNSS observables, which points to the higher complexity of the C-SVM model with potential overfitting. However, in the results of model validation by two independent datasets, the meaconing and spoofing datasets in Experiment I and II, respectively, demonstrated a high success rate and a lack of overfitting. 

Following these findings [11], we wanted to examine the effect of enriching the training data for the supervised machine learning-based approach with the real-world spoofing and meaconing datasets, eventually validating the proposed approach on these independent, real-world spoofing and meaconing datasets. In more details, we wanted to gain a deeper insight into the characteristics of the real-world spoofing and meaconing datasets, and potential implications these characteristics might have within the supervised machine learning-based approach for detection of the GNSS signal manipulation attempts. We also wanted to examine how real-world spoofing and meaconing datasets compare to the synthetically generated training datasets in terms of how successfully their usage contributes to plausible detection of real-world GNSS signal manipulation attempts. To the best of our knowledge, in this paper, we present the first attempt to train the supervised machine learning-based approach on real-world GNSS signal manipulation data. It is also the first time that a classifier constructed from the real-world GNSS manipulation data is validated on independent real-world meaconing and spoofing datasets. This gave us the ability to conduct a first-of-its-kind analysis, where deeper insights into the impacts and implications of utilizing real-world GNSS signal manipulation for the construction of a supervised machine learning-based approach are presented through comparative analysis among uniformly performed experiments. 

For this purpose, we have constructed two new experiments (Experiment III and Experiment IV). Subsequently, we examined and compared findings across all four experiments to achieve systematic insights into the transferability of the proposed approach. 

The rest of the paper is organized as follows. The next section gives an overview of the data and methods used. In this section, we only briefly describe the datasets and the methods as a more detailed description is given in Part I of this manuscript. However, we opted to present the basics for the readers, as necessary to introduce Experiment III and Experiment IV. This is followed by the results overview across both experiments presented in this paper. Next, we take a closer look and compare the obtained results with Experiments I and II introduced in Part I of the manuscript [11]. The findings across all four experiments are discussed and the main conclusions are highlighted in the final section. 

## 2. Data and Methods

### 2.1. Datasets

As mentioned in the Introduction, in this paper we have inherited the datasets described in more detail in the literature [11]. In general, we consider these to be three separated datasets:

(i) The GNSS spoofing dataset synthetically generated and radiated Over the Air (OTA) in a laboratory by means of manipulating receiver clock drift. Receiver clock drift was manipulated via Pulse Per Second (PPS) output through programmed clock divergence with an increase in the Carrier-to-Noise Density ratio (C/N_0_) of 2 dB or more for each tracked satellite (hereafter called the synthetic dataset, comprised of three subsets, each reflecting a different programmed clock divergence);
(ii) The real-world GNSS meaconing dataset produced by un-intentional re-radiation (leaked signal from laboratory) of the authentic signal (hereafter called the meaconing dataset);
(iii) The real-world spoofing dataset generated by using the Software Defined Radio (SDR) LimeSDR [12] (a low cost, open source, app-enabled SDR platform that can be used to support just about any type of wireless communication standard) and HackRF [13] (an open source hardware for SDR) configuration with gps-sdr-sim [14] (a software-defined GPS Signal Simulator that generates GPS baseband signal data streams, that can be converted to Radio-Frequency using Software-Defined Radio platforms, such as ADALM-pluto, bladeRF, HackRF, and USRP), radiated OTA and recorded by the target GNSS receiver (hereafter called the spoofing dataset).

Table 1 gives an overview of all three datasets, including specification about the number of authentic and false GNSS records within them.

### 2.2. Experiments

**Figure 1:**
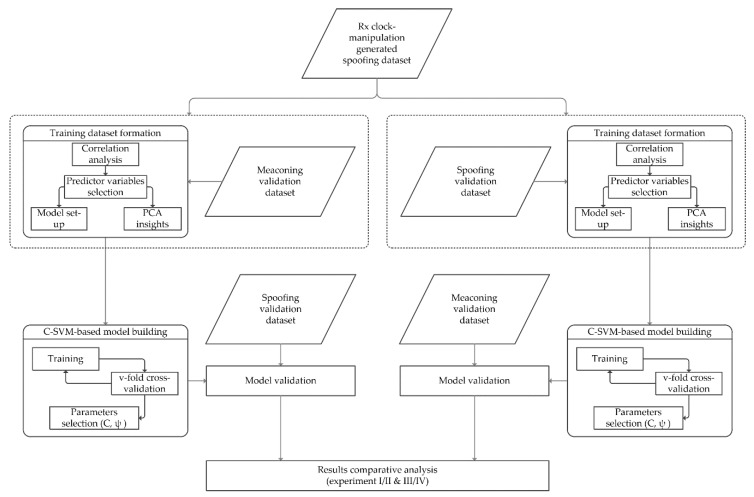
Process flow of Experiments III and IV.

In opposition to the process flow of Experiments I and II [11], training dataset formation for the supervised machine learning in Experiments III and IV was made by adding real-world meaconing and spoofing datasets to those synthetically generated GNSS datasets (based on laboratory manipulation of the GNSS receiver’s clock), respectively (hereafter called meaconing-added and spoofing added-training datasets). The following C-SVM model building was then validated interchangeably, such that the spoofing validation dataset was used for the validation of the meaconing-added training dataset, while the meaconing dataset was used for the validation of the spoofing-added training dataset (Table 2. In the final step, a comparative analysis of results obtained from Experiments I and II versus Experiments III and IV was made. The process flow of Experiments III and IV is shown in Figure 1.

### 2.3. Support Vector Machine Classification

Prior to the supervised machine learning model construction, a correlation analysis was performed [11] to support the selection of the predictor variables. Eleven variables, which have statistically significant (*p* > 0.05) correlations with the indication of whether the signal was manipulated or not (in the synthetically generated dataset) were therefore used for the C-SVM-based approach in Experiments I and II and are inherited for Experiments III and IV. These observables are:

1. Lock time [s];
2. C/N0 [0.25 dB-Hz];
3. Pseudorange [m];
4. Carrier Doppler frequency [0.0001 Hz];
5. Full carrier phase [cycles];
6. Multipath correction [0.001 m];
7. Code variance [0.0001 m2];
8. Carrier variance [mcycle2];
9. Carrier multipath correction [1/512 cycle];
10. Receiver clock bias [ms];
11. Receiver clock drift [ppm].

In the remainder of this manuscript, variables will often be referred to by their numerical order in this list. This is the case mainly for the figures and tables due to practical reasons, so as to avoid over cluttering the graphs. 

**Figure 2:**
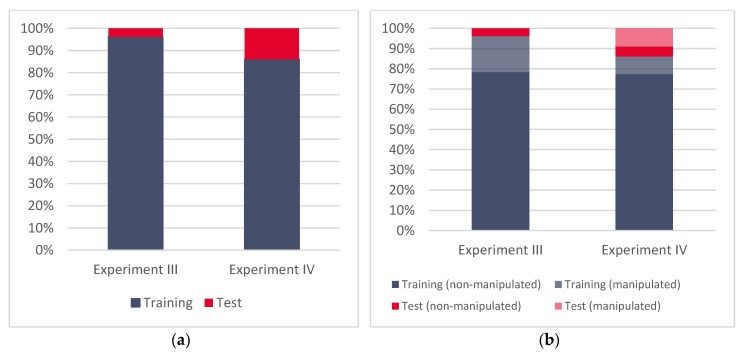
(**a**) Relative sizes of the training and test datasets for Experiment II and Experiment IV, (**b**) relative ratios of manipulated and non-manipulated Global Navigation Satellite System (GNSS) records for Experiment II and Experiment IV.

Following the correlation analysis results, we annotated the training (D_1_) and the test datasets (D_2_) separately for Experiment III and Experiment IV. For Experiment III, the training dataset included 96% of the overall data, while 4% remained and was included in the test dataset. For Experiment IV, this ratio looked a bit different (86:14) due to the differences in the sizes of the meaconing and spoofing datasets and the change in their roles in the experiments. Figure 2 shows the relative sizes of training and test (real life validation) sets across Experiment III and Experiment IV as well as the ratio of false (manipulated) and authentic (non-manipulated) records across both experiments.

Further, we have implemented C-SVM-based classification with the Radial Basis Function (RBF) kernel function [15,16,17] to transform input (predictor variables) to the feature space. Due to the relevance of capacity constants C and ψ for the C-SVM-based classification, we opted to look for the best values by utilizing the incremental grid-search as described in [11]. The range of the incremental grid search for C was from zero to 10 (with the step being equal to one) and for ψ from zero to one (with the step being equal to 0.001). The values with the best average 10-fold cross-validation accuracy were chosen to be further used on the test data. Readers interested in this approach can find more details in the literature [11,18,19].

## 3. Results

### 3.1. Experiments III and IV

Following the incremental grid search, in Experiment III the best results were achieved for ψ = 0.091 and C = 3 and, respectively, in Experiment IV, for ψ = 0.8 and C = 2. Hence, these values were used to train the C-SVM-based model. 

Looking at the overall results, in Experiment III, the C-SVM-based approach resulted in an overall success rate of 95.81% with 2000 supporting vectors (Table 3). On the other hand, Experiment IV resulted in 1706 supporting vectors and a success rate of 97.72% (Table 4). One can notice that the 10-fold cross validation success rate was slightly higher in Experiment III than the overall success rate, while in Experiment IV it was a bit lower.

**Figure 3:**
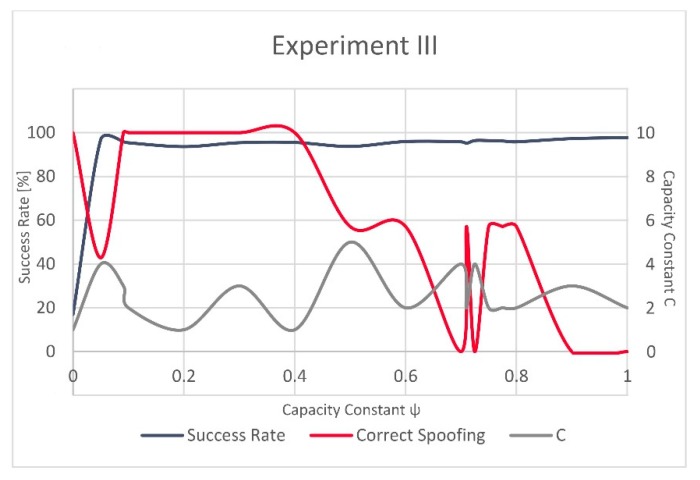
Success rate, correctly recognized spoofing records and C for Experiment III.

**Figure 4:**
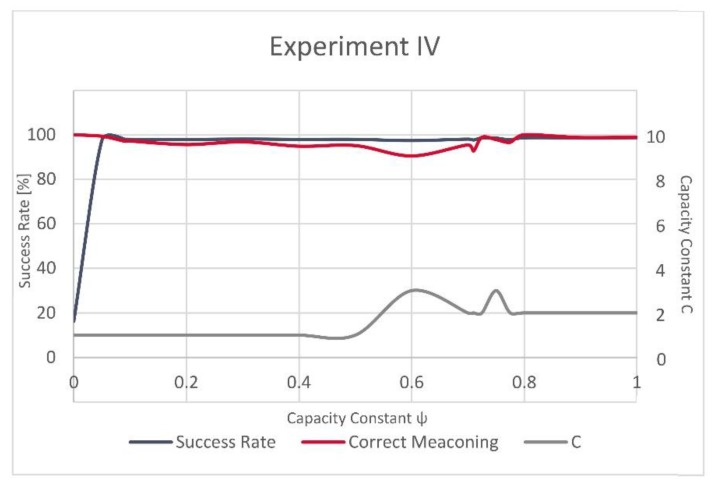
Success rate, correctly recognized meaconing records and C for Experiment IV.

Fluctuations between (i) the overall success rate for the models, (ii) the success rate of the successfully identified manipulated GNSS records (left *x*-axis), (iii) and the value of C (right *x*-axis) in relation to the different values of ψ (*y*-axis) are shown in Figure 3 for Experiment III and Figure 4 for Experiment IV. One can notice that while the overall success rate and the success rate of the correctly recognized manipulated records in Experiment IV are to some degree comparable, this is not the case with Experiment III. In Experiment III, the value of correctly recognized manipulated records is the highest between ψ = 0.1 and ψ = 0.4, followed by strong variations in the success rate and a very low value for the high ψ, while the overall success rate of the model remains high.

Table 5 presents the confusion matrix for the independent spoofing dataset used for the real-world validation in Experiment III. Respectively, Table 6 illustrates the confusion for the independent meaconing validation dataset in Experiment IV. 

### 3.2. Comparative Analysis of Findings Across Four Experiments

**Figure 5:**
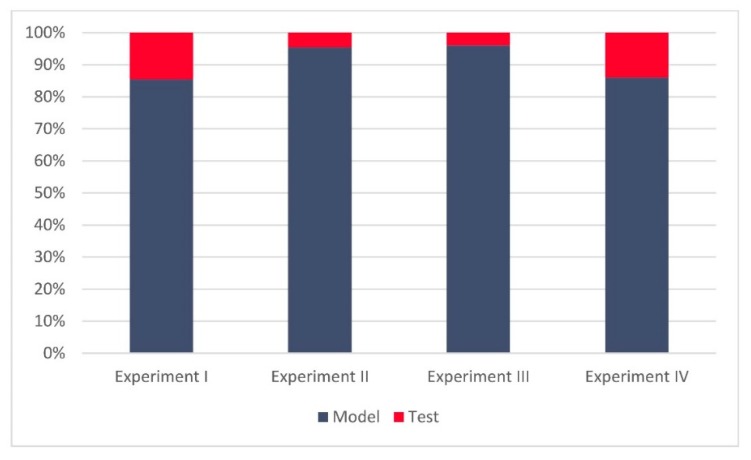
Relative sizes of the training and test datasets over all four experiments.

**Figure 6:**
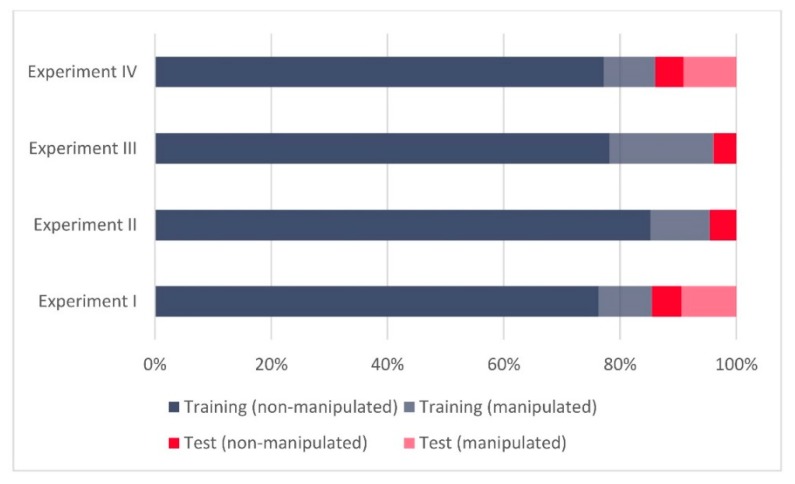
Relative ratios of manipulated and non-manipulated GNSS records in four experiments.

To gain a better insight into all four experiments and the comparability of the related findings, Figure 5 presents the relative sizes of the training and test (real-world validation) datasets over all four experiments, while Figure 6 gives more details regarding the relative ratios of manipulated and non-manipulated GNSS records. One can notice relatively comparable ratios of training and test data in Experiment I and Experiment IV, similar to those between Experiment II and Experiment III.

**Figure 7:**
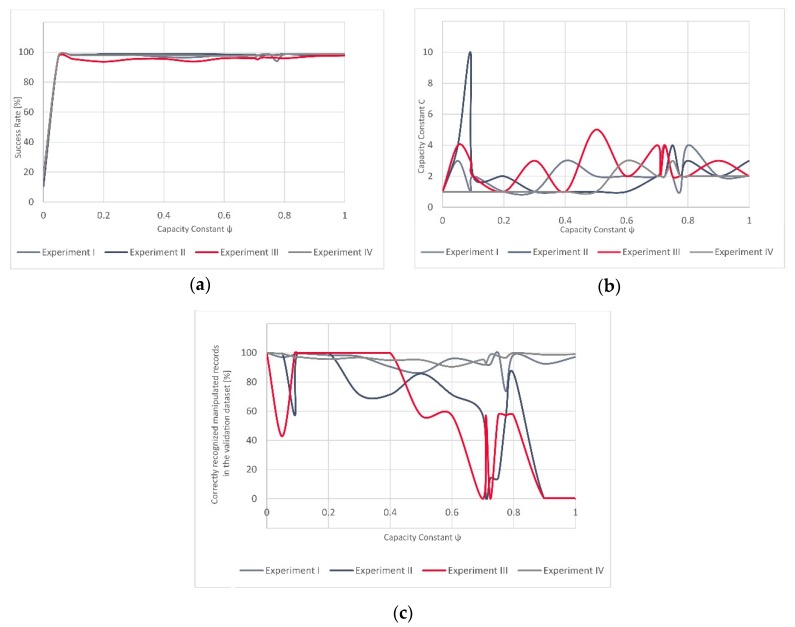
(**a**) Success rate over four experiments, (**b**) value of C over four experiments, (**c**) percentage of correctly recognized manipulated records in the validation dataset. All in relation to the ψ.

Figure 7 illustrates the development of the success rate over all four experiments, capacity constant C and the percentage of correctly recognized manipulated records in the validation dataset (all in relation to the ψ, at the horizontal *x*-axis). One can notice the relatively comparable evolution of the success rate across all four experiments. To some degree, similarities can be observed with the capacity constant C, which remains quite low over the entire ψ spectrum (with a small number of exceptions). When it comes to the number of successfully recognized manipulated records in the validation datasets, some similarities can be seen between Experiments I and IV and Experiments II and III.

Table 7 gives an overview of the best achieved results in all of the four experiments with corresponding values for capacity constrain C and *ψ*. Table 8 presents the best results globally over all four experiments for a given C and *ψ*. Globally, the best results across all four experiments were achieved for *ψ* = 0.01 and for C = 2 (mode and median values). The global success rate in such cases is 97.33%, with 96.43% correctly recognized GNSS signal manipulation attempts over all validation datasets used in all four experiments. 

**Figure 8:**
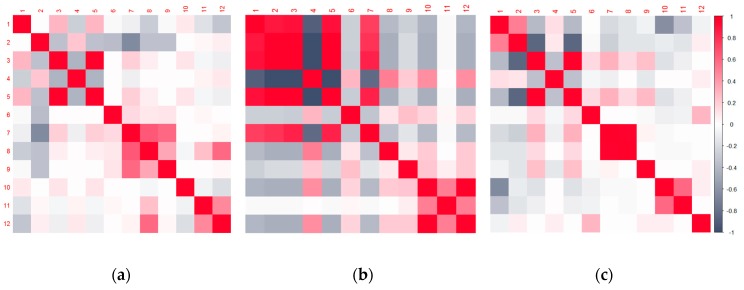
Correlation matrix for (**a**) simulated data, (**b**) meaconing and (**c**) spoofing datasets.

**Figure 9:**
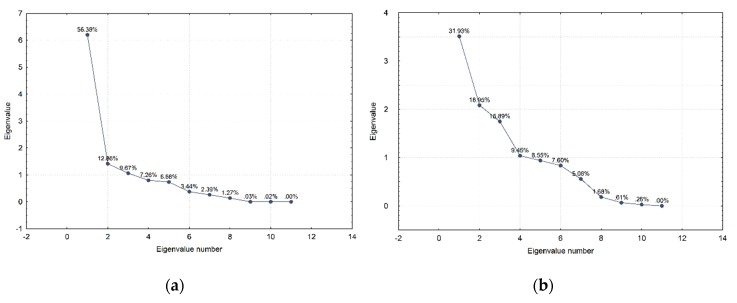

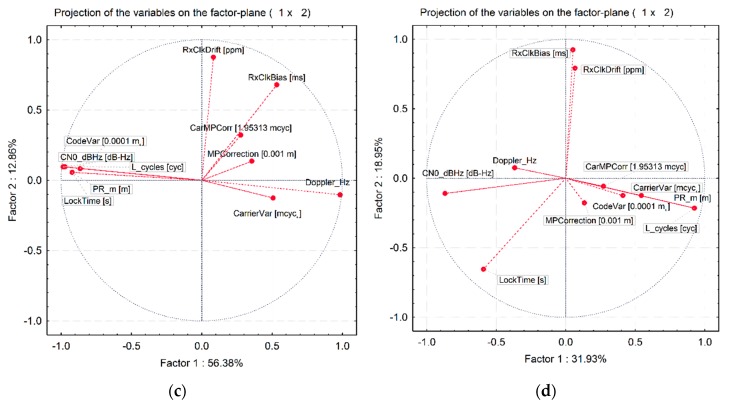
Principal components for (**a**) meaconing and (**b**) spoofing datasets and the first two factors plot for (**c**) meaconing and (**d**) spoofing datasets.

In order to better understand the context and differences in the obtained results, as well as the contribution of each dataset to these variations, we have examined the correlation patterns (Figure 8) among all three datasets. The color coding represents positive correlation values for warm colors (red spectrum) and negative correlation values for cold colors (blue spectrum). The intensity of the color represents the strength of the relationship among the observables. Hence, the strongest color intensity would be achieved for the Pearson’s correlation coefficient equal to one or −1. The numerical indication above each column, and on the left (indicating the rows), corresponds to the numerical indication of valuables, as presented in the Data and Methods section. The variable with the numerical indication twelve corresponds to the coded indication of whether the GNSS signal was manipulated or not. The coding system was zero for authentic signals and one for the manipulated ones. Furthermore, with the same motivation, we have also performed a Principal Components Analysis (PCA). Figure 9 illustrates the eigenvalues for the meaconing and spoofing datasets as well as a plot of the observables in the first two-factor coordinate system. Similar plots for the synthetic dataset can be found in Part I of this publication [11].

## 4. Discussion

Regarding the results of Experiments III and IV, one can notice that both experiments achieved relatively high success rates (95.81% and 97.72%, respectively). However, more notably, in both experiments all the GNSS signal manipulation events were successfully recognized (as reported in Table 3, Table 4, Table 5, Table 6). 

The variations in evolution of the successfully recognized GNSS signal manipulation attempts, over ψ, seem to be more stable in Experiment IV as the success rate rapidly grows for a small value of ψ and only slightly varies after this steep increase. In Experiment III, these fluctuations are more strongly visible (Figure 3 and Figure 4). However, the validation dataset in Experiment III included only a small number of the actually manipulated (spoofed) records, as failing to recognize just a few of them would result in a significant variation and would reflect in decrease of the success rate equal to almost 43%. 

The difference in the model complexity is also visible in the number of the present supporting vectors, since Experiment III resulted in 15% more supporting vectors than Experiment IV (Table 3 and Table 4). This indicates that the C-SVM-based approach found it more challenging to construct linear hyperplanes in order to correctly separate manipulated GNSS signal data points from authentic ones, in the scenario where the training dataset was composed of the simulated spoofing dataset records and those acquired from the real-world meaconing event, while the validation dataset utilized was the one created out of the real-world spoofing event records. Respectively, the linear hyperplanes for the scenario where the training set was composed of the synthetic and real-world spoofing event records, being validated on the real-world meaconing event, seemed to be a less challenging task.

In terms of the results of all four experiments, one can notice similarities in the evolution of the success rate over all four experiments (Figure 7). For the lowest value of ψ=0, the classifier always assigns only one class to all the validation dataset records (in our case the “manipulated GNSS signal class”). Hence, this starting success rate is low as the proportion of manipulated GNSS signal records, whether they were spoofing or meaconing-related, is also low. As the value of *ψ* evolves, the model reaches different success rates. Furthermore, the values of the capacity constraint C over the full spectrum of ψ values remain somewhat low, with only one peak (in Experiment II). Contrary to this, the rate of successfully recognized GNSS signal manipulation attempts fluctuates significantly. This seems to be related to the characteristics of the validation dataset. In general, the training dataset composed either of only synthetically generated data (Experiment II) or a combination of synthetically generated and real-world meaconing events (Experiment III), resulting in a C-SVM-based model that had more pronounced fluctuations when being validated on a real-world spoofing event than it was in the case of the models (Experiment I and Experiment IV) that used the real-world meaconing dataset for validation. If we consider only one value of ψ over all four experiments, then the best overall results (global results) would be achieved for a very low value of ψ = 0.01 and also very low value of C (mod and median equal to 2) (Table 7). Thus, it would be interesting in future research to see how well these values would generalize over unseen datasets (without the model training step). Such results would be particularly interesting in the context of data analytics advances, as federated learning, where models are built across multiple decentralized devices or servers holding local data samples, without exchanging the data samples. This is particularly interesting as the time-consuming training step could be avoided if the results indicated stability in such experiments. Hence, the time taken to detect a real-time sensed GNSS signal manipulation attempt would be reduced and possible mitigation strategies could be potentially developed in real-time scenarios.

To gain a better insight into the characteristics of the datasets and their impact to the training and validation roles, we have examined the principal components and the correlation patterns of the validation datasets. 

Regarding the factor analysis, or more precisely the Principal Components Analysis results for both validation datasets, one can notice that the first principal component for the real-world meaconing validation dataset accounts for 56% of variations and almost twice as many as the first principal component does in the real-world spoofing dataset (Figure 9). Furthermore, the first two principal components in the real-world meaconing validation dataset account for a comparable amount of variations to the first three principal components in the real-world spoofing dataset. This indicates that the real-world spoofing dataset has a higher number of independent variations observed in the dataset and, hence, it is more challenging to describe it by the linear combination of the variables. Looking at the component coordinates presented in the unit circle for the first two principal components, for both real-world datasets, one can notice which variables compose a linear combination that captures observed variations in the dataset and is the best represented by the current set of principal components. For the real-world meaconing dataset, the code variance, C/N_0_, full carrier phase, pseudorange, lock time, and carrier Doppler frequency compose a linear combination that is the best represented by the current set of principal components (the first component). For the real-world spoofing dataset, the first component is composed of the linear combination of C/N_0_, lock time, and full carrier phase, whereas for both datasets the second principal component captures the receiver clock bias and the receiver clock drift.

Correlation analysis (Figure 8) also returned interesting results, indicating that, to some level, the synthetically generated spoofing dataset exhibits a similar correlation pattern as the real-world meaconing dataset. However, in the real-world meaconing dataset strength of the relationship among the observables seems to be more pronounced, hence achieving the greater absolute value of Pearson’s correlation coefficient. The real-word spoofing dataset exhibits notably stronger relationships among variables, which, to some extent, differs from the patterns among the synthetically generated and the real-world meaconing datasets. For the real-world meaconing event, the strongest and most statistically significant correlation with the indication of whether the GNSS signal is manipulated or not was observed for the receiver clock bias and receiver clock drift variables. This can be interpreted by the fact that the meaconing signal is re-radiated from a distance; this is, to some extent, the same as sending it through a delay line before adding to the direct (authentic) signal. Hence, this signal delay is translated into the observed clock drift, while the exhibited variations of the clock bias in this context seem to provide the most reliable indication of the signal being manipulated or not. For the real-world spoofing dataset, the strongest and statistically significant correlation with the indication of whether the GNSS signal is manipulated or not was observed for the multipath correction variable. This can be explained by the fact that the signals in the SDR-based spoofing scenario perceive a different (and common) transfer function, so multipath correction differs from the genuine signals. Hence, the indication of whether the signal is spoofed or not is strongly reflected in the multipath correction variable correlation.

Notably, while comparing the results of the C-SVM-based approach for all four experiments, it is indicative that, after adding the real-world examples to the training dataset, the constructed models performed overall slightly worse (in Experiment III and IV) than when only synthetically generated datasets were used for the training (in Experiment I and II). However, adding the real-world generated data points to the training datasets increased the success rate of correctly recognized GNSS signal manipulation attempts. This is evident as all the real-world spoofed data points were correctly detected after the synthetically generated training dataset was enriched with the real-world meaconing data point records. This was not the case in Experiment II where the developed approach did not succeed in recognizing all the GNSS signal manipulation attempts. Hence, these results provisionally support the hypothesis that enriched training datasets with real-world-based GNSS signal manipulation records increase the supervised machine learning-based approach performance when it comes to recognizing unseen GNSS signal manipulation attempts. Nonetheless, our results indicate that they also increase the complexity of the model, as they enrich the learner with new examples that differ from those present in the synthetically generated dataset. This is an undesired effect regarding the model’s complexity.

## 5. Conclusions

In this paper, we explored the possibility of applying a supervised machine learning-based approach to detect GNSS signal manipulation attempts, particularly spoofing and meaconing attempts. To do so, we constructed four experiments (two of which are presented in Part I [11] of this publication). The two experiments presented here tackle the possibility of training the supervised machine learning-based approach on real-world GNSS signal manipulation data. We have examined and compared findings across all four experiments to achieve systematic insights into the transferability of the proposed approach. Several interesting conclusions based on our findings can be drawn.

Firstly, a supervised machine learning-based approach (in our case, C-SVM) has a high potential to be successfully implemented for GNSS signal manipulation attempt detection, as the designed model achieved high success rates over the presented experiments. Secondly, the inclusion of the real-world meaconing event increased the complexity of the model more than the inclusion of the real-world spoofing event did. This was evident in the number of supporting vectors that resulted from the model training step. However, although the increased complexity was not a desired scenario, it resulted in valuable results, as the trained model was able to detect all the real-world spoofing attempt data points in the validation step. This was not the case when the training was conducted on the simulated dataset only. Hence, enrichment of training datasets with real-world examples seems to be a valuable contribution for model creation in Safety-of-Life applications, such as the detection of GNSS signal manipulation attempts. Furthermore, if we examine all four experiments and the achieved results, there is the possibility to implement the developed approach in federated learning scenarios. This needs to be further explored in future research, but the first insights seem promising, as the values of coefficients obtained through the training in each experiment seem transferable.

## Figures and Tables

**Table 1:** Datasets.

	Laboratory Spoofing Dataset	Meaconing Dataset	Spoofing Dataset
Description	synthetically generated (simulated) data	real-world meaconing event data	real-world spoofing event data
No. of records	42636	6939	2015
Non-manipulated	38051	2531	2008
Manipulated	4585	4408	7

**Table 2:** Datasets over experiments.

	Laboratory Spoofing Dataset	Meaconing Dataset	Spoofing Dataset
Experiment I	Part of the model training	Validation data	Not included
Experiment II	Part of the model training	Not included	Validation data
Experiment III	Part of the model training	Part of the model training	Validation data
Experiment IV	Part of the model training	Validation data	Part of the model training

**Table 3:** Experiment III model summary (ψ  = 0.091, C = 3).

Experiment I	Value
Number of independents	11
SVM type	Classification type 1
Kernel type	Radial Basis Function
Number of SVs	2000 (2000 bounded)
Number of SVs (0)	1000
Number of SVs (1)	1000
Cross validation accuracy	97.97%
Class accuracy (training dataset)	96.08%
Class accuracy (independent test dataset)	89.41%
Class accuracy (overall)	95.81%

**Table 4:** Experiment IV model summary (ψ  = 0.8, C = 2).

Experiment II	Value
Number of independents	11
SVM type	Classification type 1
Kernel type	Radial Basis Function
Number of SVs	1706 (1695 bounded)
Number of SVs (0)	855
Number of SVs (1)	851
Cross validation accuracy	98.5%
Class accuracy (training dataset)	98.66%
Class accuracy (independent test dataset)	91.95%
Class accuracy (overall)	97.72%

**Table 5:** Confusion matrix for the independent spoofing validation dataset in Experiment III.

	Authentic GNSS Signal	Spoofed GNSS Signal
Authentic GNSS signal	1795	213
Spoofed GNSS signal	0	7

**Table 6:** Confusion matrix for the independent meaconing validation dataset in Experiment IV.

	Authentic GNSS Signal	Spoofed GNSS Signal
Authentic GNSS signal	1973	558
Spoofed GNSS signal	0	4408

**Table 7:** Overview of the best achieved results across all four experiments.

	Experiment I	Experiment II	Experiment III	Experiment IV
ψ	0.75	0.8	0.091	0.8
C	2	3	3	2
Success rate	98.724	98.768	95.813	97.72
Percentage of correctly recognized manipulated records in validation dataset	100	85.71	100	100

**Table 8:** Overview of the best global results over all four experiments.

	Experiment I	Experiment II	Experiment III	Experiment IV
ψ	0.01	0.01	0.01	0.01
C	2	2	2	1
Success rate	98.312	97.976	95.381	97.645
Percentage of correctly recognized manipulated records in validation dataset	100	85.71	100	100
